# CT Attenuation Thresholds for Grading Hepatic Steatosis in Adults with Overweight or Obesity and Metabolic Dysfunction-Associated Steatotic Liver Disease

**DOI:** 10.3390/diagnostics16182996

**Published:** 2026-09-16

**Authors:** Paul Góngora-Chan, Ricardo Emmanuel Jimeno-Figueroa, Dayana Williams-Jacquez, Marlene Chaurand-Lara, Ana Ligia Gutiérrez-Solis, Azalia Avila-Nava, Rodolfo Chim-Aké, Jorge Arturo Valdivieso-Jiménez, Darwin Stalin Torres-Erazo, Isabel Medina-Vera, Martha Guevara-Cruz, Roberto Lugo

**Affiliations:** 1 Departamento de Imagenología Diagnóstica y Terapéutica, Hospital Regional de Alta Especialidad de la Península de Yucatán, Servicios de Salud del Instituto Mexicano del Seguro Social para el Bienestar (IMSS-Bienestar), Mérida 97130, Mexico; medgeneral.drpaul@hotmail.com (P.G.-C.); jimeno.fig@gmail.com (R.E.J.-F.); 2 Departamento de Anatomía Patológica, Hospital Regional de Alta Especialidad de la Península de Yucatán, Servicios de Salud del Instituto Mexicano del Seguro Social para el Bienestar (IMSS-Bienestar), Mérida 97130, Mexico; williamsdayana@gmail.com; 3 Servicio de Endoscopía Digestiva, Hospital Regional de Alta Especialidad de la Península de Yucatán, Servicios de Salud del Instituto Mexicano del Seguro Social para el Bienestar (IMSS-Bienestar), Mérida 97130, Mexico; dra.chaurand@gmail.com; 4 Unidad de Investigación, Hospital Regional de Alta Especialidad de la Península de Yucatán, Servicios de Salud del Instituto Mexicano del Seguro Social para el Bienestar (IMSS-BIENESTAR), Mérida 97130, Mexico; agutierrez.hraepy@imssbienestar.gob.mx (A.L.G.-S.); zomi33@gmail.com (A.A.-N.); rodolfochim@hotmail.com (R.C.-A.); 5 Servicio de Medicina Interna, Hospital Regional de Alta Especialidad de la Península de Yucatán, Servicios de Salud del Instituto Mexicano del Seguro Social para el Bienestar (IMSS-BIENESTAR), Mérida 97130, Mexico; dr.arturo.valdivieso@gmail.com; 6 Departamento de Endocrinología, Hospital Regional Dr. Manuel Cárdenas de la Vega, Instituto de Seguridad y Servicios Sociales de los Trabajadores del Estado (ISSSTE), Culiacán 80230, Mexico; 7 Unidad de Enfermedades Infecciosas y Vigilancia Epidemiológica, Hospital Regional de Alta Especialidad de la Península de Yucatán, Servicios de Salud del Instituto Mexicano del Seguro Social para el Bienestar (IMSS-BIENESTAR), Mérida 97130, Mexico; darwintorresera@yahoo.com.mx; 8 Departamento Metodología de Investigación, Instituto Nacional de Pediatría, Ciudad de México 04530, Mexico; imedinav@pediatria.gob.mx; 9 Departamento de Fisiología de la Nutrición, Instituto Nacional de Ciencias Médicas y Nutrición Salvador Zubirán, Ciudad de México 14080, Mexico; martha.guevarac@incmnsz.mx

**Keywords:** hepatic steatosis, computed tomography, ultrasonography, MASLD

## Abstract

**Background/Objectives**: To derive computed tomography (CT) attenuation thresholds for grading hepatic steatosis and evaluate their agreement with ultrasonography (US) in adults with overweight or obesity and metabolic dysfunction-associated steatotic liver disease (MASLD) from southeastern Mexico. **Methods:** Adults (≥18 years) with MASLD who underwent both CT and US were included. Hepatic steatosis on CT was defined by liver attenuation < 40 Hounsfield units (HU) or a liver-to-spleen attenuation difference > 10 HU. US steatosis was graded using a three-point echogenicity scale. CT attenuation thresholds were derived in a training dataset (70%) using sequential receiver operating characteristic (ROC) analyses and the Youden index and were subsequently evaluated in an internal hold-out dataset (30%). Histopathological findings were available in a subgroup for additional evaluation. Agreement between CT-predicted and US-based steatosis grades was assessed using Spearman’s rank correlation coefficient (ρ) and quadratic-weighted Cohen’s kappa (κ). **Results:** In total, 105 participants were included. The training-set ROC analyses identified CT attenuation thresholds of ≤28.15 HU for Grade 3 steatosis (AUC = 0.995), 28.15–35.50 HU for Grade 2 (AUC = 0.983), and >35.50 HU for Grade 1. In the internal hold-out dataset, CT-predicted and US-based steatosis grades showed a strong positive correlation (ρ = 0.946, *p* < 0.001) and almost perfect agreement (quadratic-weighted κ = 0.940, *p* < 0.001). Liver biopsy data were available for 16 participants, and CT-predicted steatosis grade was positively associated with histological fat percentage (ρ = 0.595, *p* = 0.015). **Conclusions:** The proposed CT attenuation thresholds showed high agreement with US-based steatosis grading, and their association with histopathological fat content provided additional supportive evidence in a subgroup of participants. These findings suggest that CT attenuation may provide a useful quantitative approach to grading hepatic steatosis and could contribute to more consistent steatosis assessment across different healthcare settings.

## 1. Introduction

Metabolic dysfunction-associated steatotic liver disease (MASLD) encompasses a spectrum of hepatic and metabolic disorders characterized by abnormal accumulation of fat within hepatocytes (steatosis), generally affecting more than 5% of liver cells, in the absence of excessive alcohol consumption or other secondary causes of hepatic steatosis [1,2]. MASLD is increasingly recognized as a major public health concern because of its association with obesity, insulin resistance, type 2 diabetes mellitus, cardiovascular disease, progressive liver fibrosis, cirrhosis, and hepatocellular carcinoma.

Accurate assessment of hepatic fat accumulation is essential for the diagnosis of MASLD, evaluation of disease severity, monitoring of disease progression, and assessment of therapeutic response. Several imaging modalities are currently used to evaluate hepatic steatosis, including ultrasonography (US), magnetic resonance imaging (MRI), magnetic resonance spectroscopy, and computed tomography (CT) [3,4,5]. CT quantifies liver attenuation in Hounsfield units (HU), with lower attenuation values reflecting greater hepatic fat content [6]. US is widely used as a first-line imaging modality because of its accessibility, low cost, and lack of ionizing radiation. Hepatic steatosis is identified by increased hepatic echogenicity, with progressively impaired visualization of the intrahepatic vessels and diaphragm as disease severity increases; however, its sensitivity may be reduced in cases of mild steatosis [7]. According to conventional ultrasonographic criteria, hepatic steatosis is graded based on hepatic echogenicity and visualization of the intrahepatic vessels and diaphragm. Grade 1 is characterized by mildly increased hepatic echogenicity with preserved visualization of the vessels and diaphragm; Grade 2, by moderately increased echogenicity with partial obscuration of these structures; and Grade 3, by markedly increased echogenicity with poor or absent visualization of the intrahepatic vessels and diaphragm [8].

Although CT attenuation has long been recognized as a surrogate marker of hepatic fat accumulation, currently available attenuation thresholds for grading steatosis are heterogeneous and lack broad standardization across clinical studies and populations [6]. Most published CT criteria were originally developed to detect at least moderate hepatic steatosis rather than to stratify disease severity into clinically meaningful grades. Consequently, locally validated attenuation thresholds may improve the consistency of steatosis assessment and facilitate longitudinal follow-up in routine clinical practice. Furthermore, a standardized grading system may facilitate assessment of disease progression and therapeutic response, as well as communication between healthcare providers using different imaging modalities [9,10].

Despite the widespread use of CT and US to evaluate hepatic steatosis, few studies have simultaneously derived CT attenuation thresholds, evaluated them against histopathology, and assessed their agreement with US in routine clinical practice [11,12]. Moreover, evidence evaluating this approach in Latin American populations is limited, despite the region having one of the highest prevalences of obesity and MASLD worldwide [13].

In Mexico, obesity and metabolic disorders represent a significant healthcare burden. Approximately 36.1% of adults have obesity, and metabolic dysfunction associated with excess lipid accumulation is highly prevalent [13]. Obesity rates are particularly high in rural regions, reaching 40.2%. Populations in southeastern Mexico are exposed to dietary patterns characterized by high consumption of fats and refined carbohydrates, which contribute to insulin resistance, metabolic syndrome, and MASLD [14,15].

Additionally, in populations with limited access to high-cost medical technology, accessible and cost-effective diagnostic tools are essential for the early detection and follow-up of MASLD. In southeastern Mexico, patients are frequently referred to tertiary-care hospitals for diagnostic confirmation or specialized treatment, whereas long-term monitoring is commonly performed at community-based healthcare centers. Consequently, imaging assessments may be performed using different modalities depending on the resources available at each healthcare facility. In this context, establishing CT attenuation thresholds that provide consistent steatosis grading across CT and US is clinically relevant because these thresholds may facilitate continuity of care when patients transition between healthcare facilities.

The aim of this study was to derive CT attenuation thresholds for grading hepatic steatosis in adults with overweight or obesity and MASLD, assess their agreement with US, and explore their association with histopathological steatosis in a subgroup of participants.

## 2. Materials and Methods

### 2.1. Study Design and Population

This prospective, observational, cross-sectional study included adults (≥18 years) with suspected hepatic steatosis who were referred from the Department of Gastroenterology to the Department of Diagnostic and Therapeutic Imaging at the Hospital Regional de Alta Especialidad de la Peninsula de Yucatán, Mexico, from January to December 2024. Patients who met the eligibility criteria and agreed to participate were prospectively and consecutively enrolled during the predefined study period. Written informed consent was obtained from all participants before enrollment in the study. MASLD was diagnosed based on clinical and imaging findings, and all participants underwent both abdominal US and CT. To minimize temporal changes in hepatic fat content and ensure comparability between imaging modalities, only patients who underwent both examinations within a maximum interval of 2 days were included.

The exclusion criteria were alcohol consumption of >30 g/day in men or >20 g/day in women; active malignancy requiring chemotherapy or radiotherapy; infection with human immunodeficiency virus, hepatitis B virus, or hepatitis C virus; established cirrhosis; pregnancy; and use of medications associated with drug-induced liver injury.

Sociodemographic and clinical variables collected included age, sex, place of residence, height, weight, body mass index (BMI), and metabolic comorbidities associated with MASLD.

### 2.2. CT Assessment of Hepatic Steatosis

Abdominal noncontrast CT examinations were performed using a 64-detector-row scanner (Revolution EVO; GE Healthcare, Milwaukee, WI, USA). Imaging parameters included a slice thickness of 5 mm, tube voltage of 120 kVp, and tube current of 180 mA. All examinations were performed without intravenous contrast administration.

CT images were independently analyzed by two board-certified radiologists using RadiAnt DICOM Viewer (version 4.6.4) to improve the reliability of attenuation measurements. A single circular region of interest (ROI) of approximately 200 mm^2^ or larger was placed in homogeneous hepatic parenchyma, generally in segment VII of the right hepatic lobe, avoiding major blood vessels, biliary structures, focal lesions, and other structures that could affect attenuation measurements. An additional ROI was placed in homogeneous splenic parenchyma to calculate the liver-to-spleen attenuation difference. Mean attenuation values were recorded in HU.

For the initial CT classification, moderate-to-severe hepatic steatosis was defined as a liver attenuation value of <40 HU or a liver-to-spleen attenuation difference of >10 HU, according to previously validated CT criteria [16,17]. CT attenuation thresholds for grading hepatic steatosis were subsequently derived from the study population using receiver operating characteristic (ROC) analysis and the Youden index to identify optimal cutoff values.

### 2.3. Ultrasonographic Assessment of Hepatic Steatosis

Ultrasonographic examinations were performed using a Philips Affiniti 70 ultrasound system (Philips Ultrasound Inc., Bothess, WA, USA) equipped with a 5- to 10 MHz convex transducer. Conventional B-mode US was used for all examinations, following a standardized scanning protocol that evaluated both hepatic lobes.

Hepatic steatosis was assessed based on hepatic echogenicity relative to the walls of the portal vein and intrahepatic bile ducts, as well as visualization of the diaphragm. Steatosis severity was graded according to established qualitative B-mode ultrasonographic criteria [8,18]. Conventional B-mode US grading was selected as the imaging reference classification because of its widespread availability and established role as a first-line imaging approach for assessing hepatic steatosis in routine clinical practice. Grade 1 (mild) was defined as a slight increase in hepatic echogenicity with preserved visualization of the portal vein walls, bile duct walls, and diaphragm; Grade 2 (moderate) was defined as a moderate increase in hepatic echogenicity with reduced visualization of the portal vein and bile duct walls, while the diaphragm remained visible; and Grade 3 (severe) was defined as a marked increase in hepatic echogenicity with loss of visualization of the portal vein and bile duct walls and poor visualization of the diaphragm.

US examinations were independently interpreted by two board-certified radiologists using conventional B-mode imaging.

### 2.4. Interobserver and Intraobserver Imaging Assessment

CT and US examinations were independently interpreted by two board-certified radiologists with more than 6 years of experience in diagnostic imaging. During image interpretation, the radiologists did not have access to the participants’ clinical information or to the other radiologist’s measurements or classifications.

Interobserver reproducibility was assessed using the independent evaluations performed by both radiologists for the entire study cohort (*n* = 105). To assess intraobserver reproducibility, approximately one-third of the cohort (35/105 participants) was selected for repeat imaging assessment, with representation across all three steatosis grades. More than 4 months after the initial assessment, both radiologists independently reassessed the CT and US images using the same evaluation procedures. During reassessment, the radiologists reviewed only the imaging studies and had no access to the participants’ clinical records or their previous measurements and classifications. For intraobserver reproducibility, the repeated measurements and classifications of each radiologist were compared with their respective initial assessments.

For CT threshold derivation and internal hold-out evaluation, attenuation measurements from Radiologist 2 were used. For US, concordant grades from both radiologists were used; in cases of disagreement, both radiologists jointly reviewed the images and reached a consensus based on hepatic echogenicity and the visualization of anatomical structures, including intrahepatic vessels and the diaphragm, across the available imaging planes. The same approach was applied to both the training and internal hold-out datasets.

### 2.5. Histopathological Assessment

A subgroup of participants with hepatic steatosis and concomitant clinical suspicion of hepatic neoplasia underwent ultrasound-guided Tru-Cut liver biopsy as part of routine clinical care. Biopsies were performed based on clinical indications and were not required by the study protocol. Tissue specimens were fixed in formalin, embedded in paraffin, sectioned, and stained with hematoxylin and eosin according to standard histopathological procedures.

Histological assessment was performed by a board-certified pathologist with expertise in liver pathology who was blinded to the patients’ clinical information and both CT and US findings. Hepatic steatosis was quantified as the percentage of hepatocytes containing macrovesicular fat vacuoles and categorized as normal (<5%), mild (5–33%), moderate (34–65%), or severe (>66%) [19].

### 2.6. Development and Internal Hold-Out Evaluation of CT Attenuation Thresholds for Steatosis Grading

To minimize overfitting, the study population was randomly divided into a training dataset (70%) and an internal hold-out dataset (30%) using a fixed random seed. CT attenuation thresholds for grading hepatic steatosis were derived exclusively from the training dataset.

Two sequential binary ROC analyses were performed using US steatosis grades as the reference classification. The first model discriminated Grade 1 from Grades 2–3 steatosis, whereas the second discriminated Grades 1–2 from Grade 3 steatosis. Optimal attenuation thresholds were identified using the Youden index. Areas under the ROC curve (AUCs) and corresponding 95% confidence intervals (CIs) were calculated for each model. The derived attenuation thresholds were then applied to the internal hold-out dataset to generate CT-predicted steatosis grades.

### 2.7. Statistical Analysis

Categorical variables were expressed as frequencies and percentages, whereas continuous variables were expressed as mean ± standard deviation or median with interquartile range [Q1–Q3], as appropriate. There were no indeterminate CT or US results or missing imaging data among the included participants; therefore, all participants were included in the imaging analyses.

Interobserver reliability of CT attenuation measurements between the two radiologists was assessed using the intraclass correlation coefficient (ICC), based on a two-way random-effects model for absolute agreement and single measurements [ICC (2,1)]. Interobserver agreement for US steatosis grading was assessed using quadratic-weighted Cohen’s kappa (κ), given the ordinal nature of the three steatosis grades. Intraobserver reliability was also evaluated in the subset of 35 participants who underwent repeat assessments by both radiologists, using the corresponding statistical approach for CT and US. Ninety-five percent CIs for reliability coefficients were estimated using bootstrap resampling at the participant level.

Agreement between CT-predicted and US steatosis grades in the internal hold-out dataset was assessed using Spearman’s rank correlation coefficient (ρ), Kendall’s tau (τ), and quadratic-weighted Cohen’s kappa (κ). Kappa coefficients were interpreted according to the classification proposed by Landis and Koch.

Histopathological assessment was performed in the subgroup of participants with available liver biopsy data. Given the limited size of this subgroup, the histopathological analyses were considered exploratory. Histological fat percentage was summarized according to CT-predicted steatosis grade, and the association between histological fat percentage and CT-predicted grade was assessed using Spearman’s rank correlation coefficient.

All statistical analyses were performed using R software (version 4.5.3). Statistical significance was defined as a two-sided *p* value of <0.05.

## 3. Results

### 3.1. Participant Characteristics

**Figure 1:**
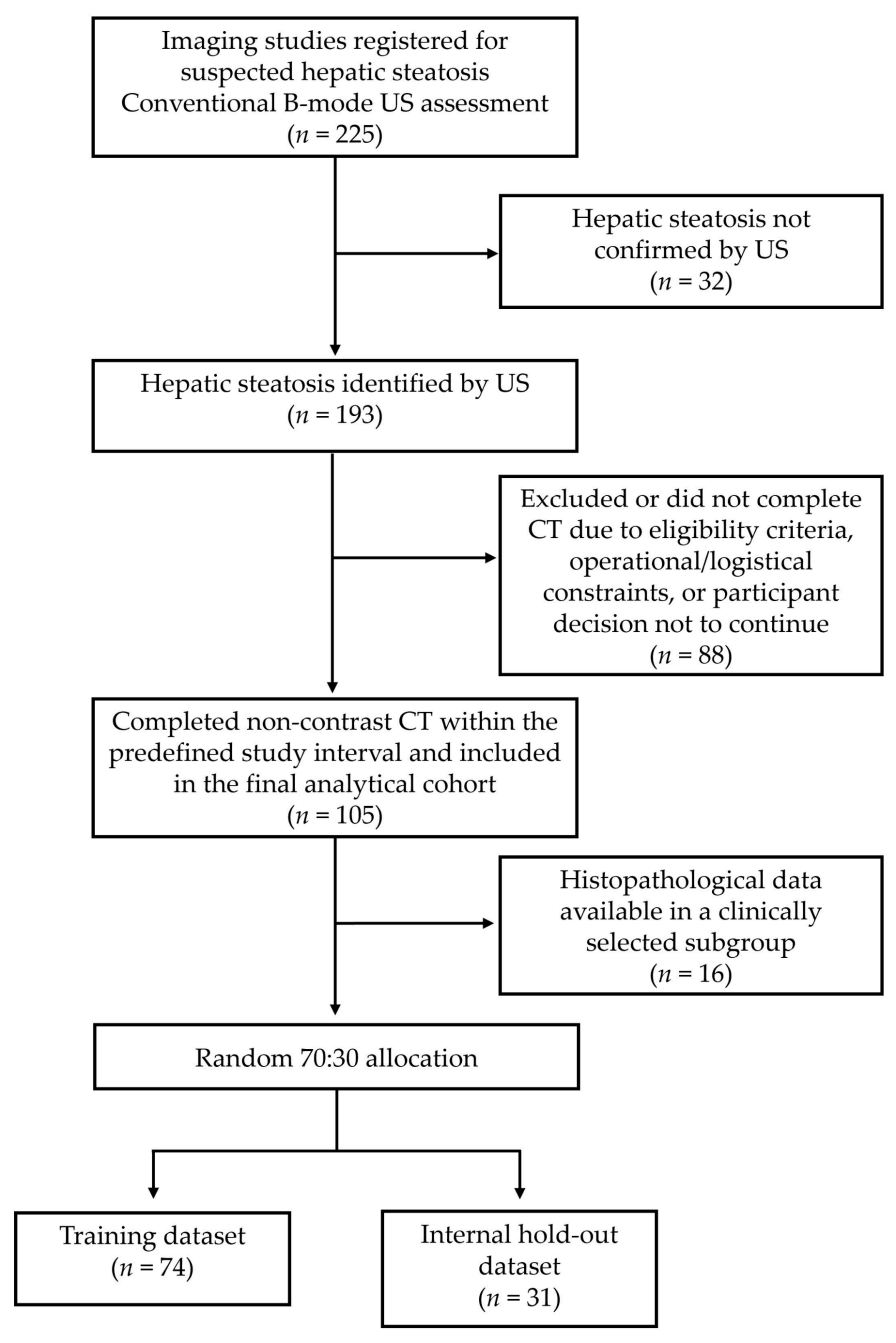
Standards for Reporting Diagnostics Accuracy Studies (STARD) flow diagram of participant inclusion and study analyses. CT: computed tomography; US: ultrasonography.

In total, 105 participants were included in the final analytical cohort after application of the eligibility criteria (Figure 1). The cohort comprised 38 men (36.2%) and 67 women (63.8%). Their median age was 42 years [33.0–51.0], with no significant difference between men and women. The median BMI was 33.1 [29.7–36.3] kg/m^2^, and 75 participants (71.4%) had obesity. Metabolic comorbidities were common, particularly type 2 diabetes (31.4%), hypertension (30.5%), and dyslipidemia (29.5%). Overall, 89% of participants had at least one additional metabolic comorbidity associated with MASLD. Detailed demographic and clinical characteristics are presented in Table 1.

### 3.2. Interobserver and Intraobserver Reliability

Interobserver reliability for CT attenuation measurements was excellent (ICC = 0.944; 95% CI, 0.900–0.967). Among the 35 participants who underwent repeat assessment, intraobserver reliability was good for Radiologist 1 (ICC = 0.874; 95% CI, 0.707–0.954) and excellent for Radiologist 2 (ICC = 0.942; 95% CI, 0.875–0.971). Interobserver agreement for US steatosis grading was also high (quadratic-weighted κ = 0.899; 95% CI, 0.823–0.953), with similarly high intraobserver agreement for Radiologist 1 (κ = 0.871; 95% CI, 0.730–0.968) and Radiologist 2 (κ = 0.892; 95% CI, 0.749–0.977) (Table 2).

### 3.3. Derivation of CT Attenuation Thresholds for Steatosis Grading

Two sequential ROC analyses were performed in the training dataset (*n* = 74) to derive optimal CT attenuation thresholds for steatosis grading. The first ROC analysis, distinguishing Grade 1 from Grade ≥ 2 steatosis, identified an optimal threshold of 35.50 HU. The second ROC analysis, distinguishing Grade ≤ 2 from Grade 3 steatosis, identified an optimal threshold of 28.15 HU.

Based on these thresholds, CT-predicted hepatic steatosis was classified as Grade 1 (>35.50 HU), Grade 2 (28.15–35.50 HU), or Grade 3 (≤28.15 HU). In the training dataset, the two ROC analyses showed high discriminatory performance, with AUCs of 0.983 (95% CI, 0.963–1.000) and 0.995 (95% CI, 0.986–1.000), respectively. These estimates reflect performance within the training dataset used for threshold derivation and should therefore be distinguished from performance in the internal hold-out dataset. Detailed training-set ROC metrics are presented in Table 3, and the corresponding ROC curves are shown in Supplementary Figure S1.

### 3.4. Internal Hold-Out Evaluation of CT Attenuation Thresholds Using Ultrasonography

The training (*n* = 74) and internal hold-out (*n* = 31) datasets included participants across all three US steatosis grades; their distributions are summarized in Table 4. The derived CT attenuation thresholds were then applied to the internal hold-out dataset. Overall classification accuracy was 93.5%, with 29 of 31 participants correctly classified. The two misclassifications involved only adjacent steatosis grades: one Grade 1 case was classified as Grade 2, and one Grade 2 case was classified as Grade 3. No misclassifications between nonadjacent grades were observed.

**Figure 2:**
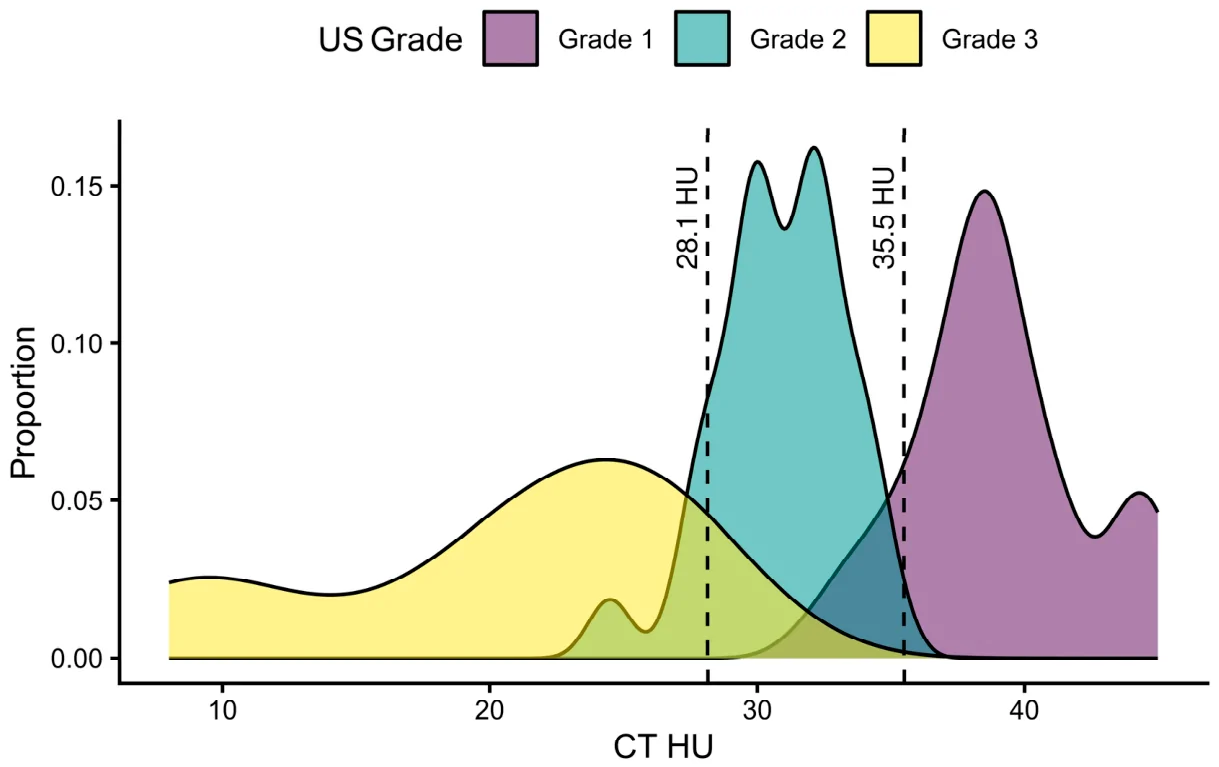
Kernel density distributions of CT attenuation values according to US-defined steatosis grade in the full cohort (*n* = 105). Dashed vertical lines indicate the optimal cutpoints derived exclusively from the training dataset (*n* = 74) using the Youden index: 28.15 HU (Grade ≤ 2 vs. Grade 3) and 35.50 HU (Grade 1 vs. Grade ≥ 2). CT: Computed tomography; HU: Hounsfield units.

Agreement between CT-predicted and US steatosis grades was high across the ordinal association and agreement measures (Table 5). Spearman’s rank correlation coefficient showed a very strong positive association (ρ = 0.946, *p* < 0.001), corroborated by Kendall’s tau (τ = 0.930, *p* < 0.001). Quadratic-weighted Cohen’s κ indicated almost perfect agreement (κ = 0.940, *p* < 0.001). The distribution of CT attenuation values by US steatosis grade is shown in Figure 2.

**Figure 3:**
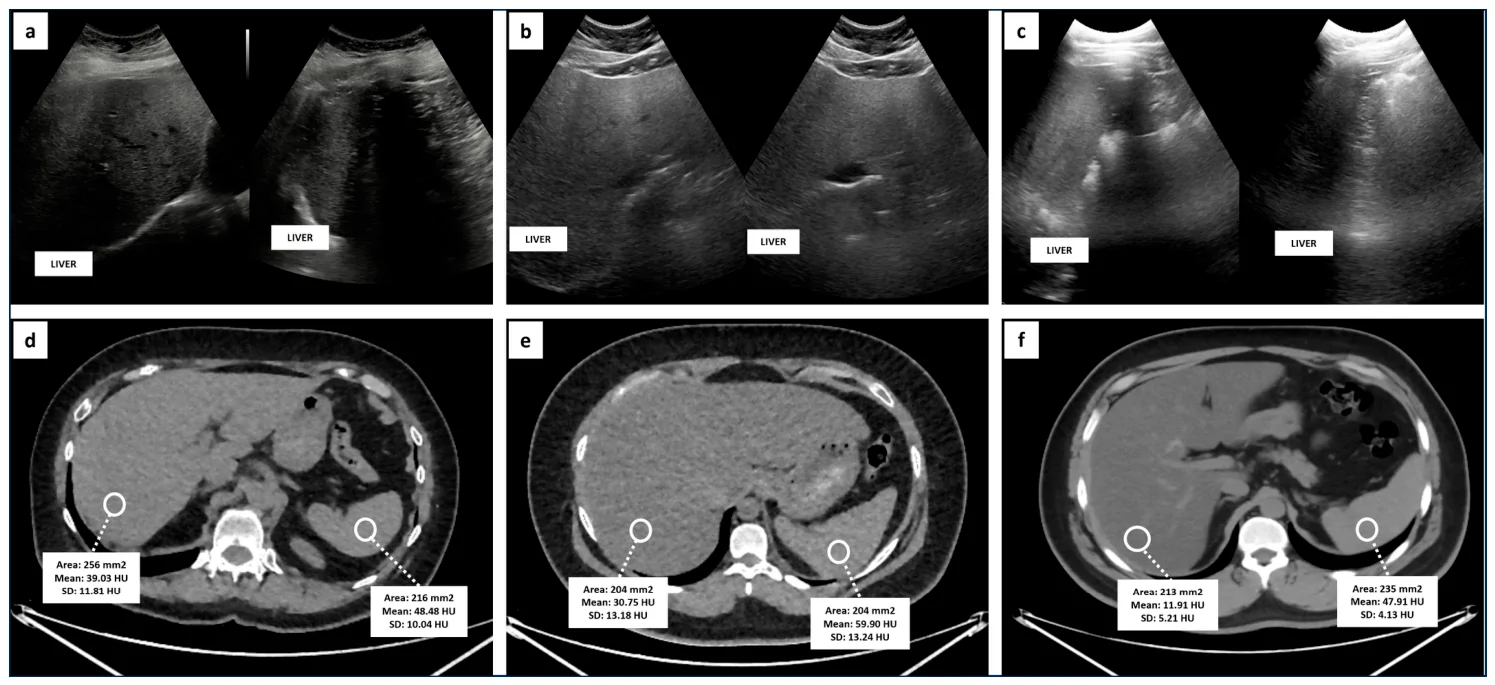
Representative B-mode ultrasonographic and non-contrast CT images illustrating the three grades of hepatic steatosis. US images show (**a**) Grade 1, (**b**) Grade 2, (**c**) Grade 3 steatosis. Corresponding CT images show hepatic and splenic attenuation values of (**d**) 39.03 and 48.48 HU, (**e**) 30.75 and 59.90 HU, and (**f**) 11.91 and 47.91 HU, respectively. Hepatic and splenic ROIs used for attenuation measurements are indicated on the CT images. CT: Computed tomography; US: Ultrasonography; ROI: Region of interest.

Representative CT and US images illustrating the different grades of hepatic steatosis are presented in Figure 3.

### 3.5. Histopathological Validation in a Subgroup

**Figure 4:**
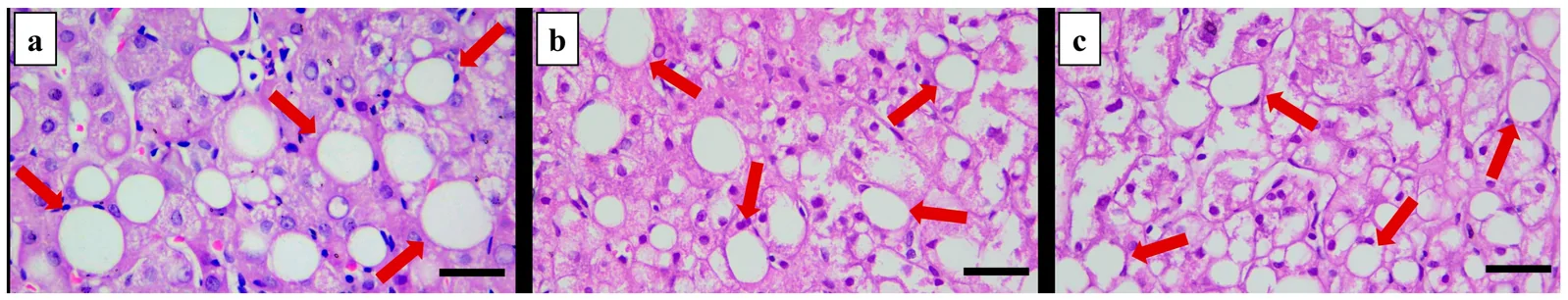
Representative histological images showing different degrees of hepatic steatosis: (**a**) mild steatosis, with approximately 20% of hepatocytes affected; (**b**) moderate steatosis, with approximately 40% of hepatocytes affected; and (**c**) severe steatosis, with approximately 80% of hepatocytes affected. Hematoxylin and eosin staining, 40× magnification. Scale bar 50 µm. Red arrows indicate hepatocytes containing macrovesicular fat vacuoles.

Liver biopsy data were available for 16 participants. Histological fat content increased progressively across CT-predicted steatosis grades. Mean histological fat content was 18.0% ± 5.7% for Grade 1, 34.0% ± 21.0% for Grade 2, and 69.3% ± 33.9% for Grade 3. A significant positive correlation was observed between CT-predicted steatosis grade and histological fat percentage (Spearman’s ρ = 0.595, *p* = 0.015). Representative histopathological images showing mild, moderate, and severe steatosis are presented in Figure 4.

## 4. Discussion

The present study derived CT attenuation thresholds for grading hepatic steatosis in adults with overweight or obesity and MASLD from southeastern Mexico and evaluated their performance in an internal hold-out dataset. To our knowledge, this is the first study from southeastern Mexico to derive CT attenuation thresholds for a three-grade classification of hepatic steatosis. The proposed thresholds showed high agreement with US steatosis grading, and CT-predicted steatosis grades were positively associated with histopathological fat content in a subgroup of participants. These findings support the potential use of CT attenuation as a quantitative approach to grading hepatic steatosis.

The prevalence of obesity and metabolic disorders is particularly high in southeastern Mexico, including the Yucatan Peninsula, where central obesity, insulin resistance, and other metabolic risk factors contribute substantially to the burden of MASLD [13,14,15]. In this setting, patients are frequently evaluated at different levels of care, with varying access to imaging modalities. Although CT is commonly available in tertiary-care hospitals, US remains the principal imaging modality in community healthcare centers. Consequently, standardized imaging criteria that enable comparable steatosis grading across modalities could facilitate continuity of care when different imaging resources are available.

These regional epidemiological characteristics were reflected in the clinical profile of our study population, which had a substantial cardiometabolic burden, characterized by a high prevalence of obesity, type 2 diabetes, hypertension, and dyslipidemia. In our cohort, obesity was more frequent among women, whereas men had higher systolic blood pressure. These findings are consistent with previous reports indicating that excess adiposity, insulin resistance, and cardiometabolic disorders are major determinants of the development and progression of MASLD [13,20,21]. Likewise, the elevated BMI in our cohort is consistent with studies demonstrating that increasing adiposity is strongly associated with hepatic fat accumulation and a higher prevalence of MASLD [22,23]. Previous studies in Mexican adults have also linked excess adiposity and central lipid accumulation to increased oxidative stress, further supporting the complex metabolic alterations associated with overweight and obesity in this population [24,25]. Almost 9 out of 10 participants had at least one additional metabolic comorbidity, highlighting the substantial cardiometabolic burden in this cohort.

Interestingly, nearly half of the participants had a history of urolithiasis. Although this variable was not associated with CT-based steatosis grading in the present study, its high prevalence further reflects the complex metabolic profile of this cohort. Recent evidence indicates that MASLD is associated with an increased risk of nephrolithiasis, likely because the two conditions share pathogenic mechanisms, including insulin resistance, obesity, chronic low-grade inflammation, and metabolic dysregulation [26]. Moreover, studies conducted in southeastern Mexico have reported a high burden of recurrent urolithiasis, further supporting the relevance of this finding in our regional population [27,28].

Although liver biopsy remains the reference standard for assessing hepatic steatosis, its invasive nature, sampling variability, and potential complications limit its routine use in clinical practice [2,10]. MRI-based techniques, particularly MRI-derived proton density fat fraction (MRI-PDFF), provide accurate quantitative measurements of hepatic fat and have shown strong correlations with histological steatosis [5,29,30,31]. MRI techniques may also allow regional or volumetric assessment of hepatic fat distribution, providing a more comprehensive characterization of hepatic steatosis. However, their cost and limited availability may restrict their routine use in some healthcare settings [32,33]. Thus, noncontrast CT and US remain clinically relevant imaging modalities for the assessment and follow-up of MASLD, particularly in resource-constrained healthcare systems [17,31]. CT provides quantitative measurements of hepatic attenuation in HU, whereas conventional B-mode US provides a qualitative assessment based on characteristic changes in hepatic echogenicity and visualization of intrahepatic structures [7,34].

Regional ROI-based measurements provide a simple approach to estimating hepatic fat content, although they may not fully capture spatial heterogeneity in hepatic fat distribution. In contrast, volumetric MRI-based approaches, particularly chemical shift-encoded MRI (CSE-MRI) with PDFF mapping, enable assessment of fat content across the entire liver and may therefore provide a more comprehensive characterization of hepatic steatosis [32]. Similarly, recent CT-based approaches incorporating automated liver and spleen attenuation measurements and volumetric segmentation have been developed to provide a more comprehensive assessment of hepatic attenuation [35,36]. In the present study, CT attenuation was measured using a single hepatic ROI of approximately 200 mm^2^, generally placed in segment VII of the right hepatic lobe under standardized conditions. Although this approach showed high reproducibility between radiologists, as demonstrated by the interobserver reliability in our study, regional attenuation measurements should be interpreted in light of the potential heterogeneity of hepatic fat distribution.

The present study identified CT attenuation thresholds of 35.50 HU and 28.15 HU for differentiating mild, moderate, and severe hepatic steatosis. Previous CT studies have primarily proposed single attenuation thresholds, commonly ranging from 40 to 45 HU, liver-to-spleen attenuation differences, or liver-to-spleen attenuation ratios to identify at least moderate hepatic steatosis rather than to grade disease severity [37,38]. The absence of universally accepted CT thresholds likely reflects differences in study populations, reference standards, acquisition protocols, and analytical approaches [6]. By contrast, our study used two sequential ROC analyses to derive thresholds for classification into three clinically relevant steatosis grades. However, the high AUCs observed during threshold derivation should be interpreted cautiously because discriminatory performance was estimated in the same training dataset used to identify the optimal cutpoints and may therefore be optimistic. The thresholds were subsequently evaluated in a separate internal hold-out dataset, as discussed below. Although 35.50 and 28.15 HU represent the statistically derived optimal cutpoints, rounded values may be more practical for clinical interpretation; however, their diagnostic performance should be specifically evaluated before their adoption as operational thresholds.

When applied to the internal hold-out dataset, the derived CT attenuation thresholds correctly classified 29 of 31 participants (93.5%) according to US steatosis grade. Agreement between CT-predicted and US-defined steatosis grades was high, as demonstrated by Spearman’s correlation (ρ = 0.946), Kendall’s tau (τ = 0.930), and quadratic-weighted Cohen’s κ (κ = 0.940). Our findings are consistent with previous studies reporting good agreement between CT and US in the evaluation of hepatic steatosis, particularly for moderate-to-severe disease [6,37,39]. Importantly, the two observed misclassifications occurred only between adjacent steatosis grades, with no misclassification between mild and severe steatosis. These findings support the consistency of the proposed thresholds with US grading; however, they should be interpreted cautiously given the relatively small internal hold-out dataset (*n* = 31).

An important consideration is that the CT attenuation thresholds were derived using conventional B-mode US grading as the imaging reference and should therefore be interpreted as US-derived CT thresholds rather than histologically established cutpoints. Although conventional B-mode US is inherently qualitative and operator-dependent, steatosis grading in the present study was based on systematic evaluation of predefined imaging features, including hepatic echogenicity and visualization of the intrahepatic vascular structures and diaphragm. In addition, all examinations were performed using the same ultrasound system and standardized acquisition procedures, which may have reduced equipment-related variability. The high interobserver (quadratic-weighted κ = 0.899) and intraobserver agreement (κ = 0.871 and 0.892) observed in our study further supports the consistency of the US grading procedure. More recently, quantitative ultrasound-based techniques have demonstrated high diagnostic performance for hepatic fat assessment compared with MRI-PDFF and histological reference standards [40]. These approaches may overcome some limitations inherent to conventional qualitative B-mode grading and may serve as relevant reference methods for future validation of the proposed CT thresholds.

Histopathological validation in the subgroup of 16 participants provided additional evidence supporting the relationship between CT-predicted steatosis grade and hepatic fat content. Histological fat percentage increased progressively across CT-predicted grades, and a significant positive correlation was observed between CT-predicted steatosis grade and histological fat percentage. These findings are consistent with previous studies showing that decreasing liver attenuation on noncontrast CT is associated with increasing hepatic fat accumulation and correlates with histological steatosis severity [11,16,37]. However, given the small size and clinically selected nature of the biopsy subgroup, these findings should be considered exploratory and interpreted as supportive rather than definitive histopathological validation of the proposed thresholds.

The present findings may have relevant clinical implications in southeastern Mexico, where patients with MASLD may be evaluated at different levels of care with considerable variation in imaging availability. The high agreement between CT-predicted and US steatosis grades suggests that the proposed CT attenuation thresholds may provide a complementary approach to grading hepatic steatosis when CT is clinically available. These findings provide region-specific evidence on the use of CT attenuation for three-grade classification of hepatic steatosis in this population. However, the application of these thresholds across different healthcare settings and for longitudinal assessment requires further evaluation in prospective, multicenter studies.

This study has several strengths, including its prospective design, standardized CT and US acquisition procedures, and use of separate datasets for threshold derivation and internal hold-out evaluation. Imaging assessments were performed independently by two experienced radiologists, with additional evaluation of interobserver and intraobserver reproducibility. Furthermore, the availability of histopathological data for a subgroup of participants provided complementary evidence of the relationship between CT-predicted steatosis grade and hepatic fat content.

However, several limitations should be acknowledged. First, the study was conducted at a single tertiary-care referral hospital, and the internal hold-out dataset was relatively small. Although this approach allowed the derived thresholds to be evaluated in participants who were not included in the training dataset, their generalizability to other populations and healthcare settings remains to be established. Second, histopathological assessment was available only for a small, clinically selected subgroup of participants who underwent liver biopsy based on clinical indications. Therefore, these findings should be considered supportive and exploratory, and potential selection bias cannot be excluded. Third, CT attenuation was assessed using a single hepatic ROI under standardized conditions. Although this approach provided reproducible attenuation measurements, a single regional measurement may not fully capture heterogeneous fat distribution throughout the liver. In addition, complete reciprocal blinding between CT and US was not possible because CT examinations were performed after US identification of hepatic steatosis as part of the recruitment pathway, which may have introduced review bias. Fourth, all CT examinations were performed using the same scanner and acquisition protocol, which improved methodological consistency but may limit the transferability of the proposed thresholds to other CT systems and imaging protocols. Finally, the study population consisted of adults with overweight or obesity and MASLD, and differences in body habitus may affect CT image quality and measurement variability. Therefore, larger multicenter studies including more diverse populations, different CT systems, and external validation cohorts are warranted to confirm and refine the proposed attenuation thresholds before broader clinical implementation.

## 5. Conclusions

The present study derived CT attenuation thresholds for grading hepatic steatosis in adults with overweight or obesity and MASLD from southeastern Mexico and evaluated their performance in an internal hold-out dataset. The proposed thresholds showed high agreement with US steatosis grading, and the positive association between CT-predicted steatosis grade and histopathological fat content in a subgroup of participants provided additional support for the proposed classification. These findings suggest that CT attenuation may provide a useful quantitative approach to grading hepatic steatosis and could facilitate more consistent assessment across different levels of healthcare with varying imaging resources, particularly in resource-constrained healthcare systems.

## Figures and Tables

**Table 1:** Demographic, anthropometric, and clinical characteristics of the study population.

Characteristics	Total *n* = 105	Men *n* = 38	Women *n* = 67
Age in years	42.0 [33.0–51.0]	40.0 [33.0–51.0]	42.5 [33.0–51.2]
Age in years (min–max)	19–81	19–61	21–81
Place of birth, *n* (%):			
Yucatan	97 (92.4)	34 (32.4)	63 (60.0)
Campeche	4 (3.8)	2 (1.9)	2 (1.9)
Quintana Roo	4 (3.8)	2 (1.9)	2 (1.9)
Anthropometric characteristics			
Height (m)	1.59 [1.53–1.65]	1.59 [1.51–1.65]	1.57 [1.52–1.62]
Weight (kg)	84.0 [71.9–93.0]	84.0 [72.0–95.0]	83.4 [71.0–93.0]
BMI (kg/m^2^)	33.1 [29.7–36.3]	34.1 [30.4–38.3]	33.0 [29.8–36.2]
BMI classification, *n* (%):			
Overweight	30 (28.6)	10 (9.5)	20 (19.1)
Obesity class I	30 (28.6)	15 (14.3)	15 (14.3)
Obesity class II	35 (33.3)	10 (9.5)	25 (23.8)
Obesity class III	10 (9.5)	3 (2.9)	7 (6.6)
SBP (mm Hg)	120 [110–130]	123 [115–137]	118 [110–124]
DBP (mm Hg)	80.0 [72.8–87.8]	81.5 [76.2–88.5]	79.7 [71.3–86.6]
Conditions associated with the diseases, *n* (%):			
Type 2 diabetes	33 (31.4)	11 (10.4)	22 (21.0)
Hypertension	32 (30.5)	12 (11.4)	20 (19.1)
Dyslipidemia	31 (29.5)	12 (11.4)	19 (18.1)
Urolithiasis	52 (49.5)	17 (16.2)	35 (33.3)
Chronic kidney disease	9 (8.5)	2 (1.9)	7 (6.6)
None	25 (23.8)	10 (9.5)	15 (14.3)

Data were expressed in the median and interquartile [Q1–Q3] or frequency and percentage, n (%). BMI: body mass index; SBP: Systolic blood pressure; DBP: Diastolic blood pressure.

**Table 2:** Interobserver and intraobserver reliability of CT attenuation measurements and US steatosis grading.

Modality	Reliability Assessment	*n*	Statistic	Estimate	95% CI
CT attenuation	Interobserver, Radiologist 1 vs. Radiologist 2	105	ICC (2,1)	0.944	0.900–0.967
CT attenuation	Intraobserver, Radiologist 1	35	ICC (2,1)	0.874	0.707–0.954
CT attenuation	Intraobserver, Radiologist 2	35	ICC (2,1)	0.942	0.875–0.971
US steatosis grade	Interobserver, Radiologist 1 vs. Radiologist 2	105	Weighted κ	0.899	0.823–0.953
US steatosis grade	Intraobserver, Radiologist 1	35	Weighted κ	0.871	0.730–0.968
US steatosis grade	Intraobserver, Radiologist 2	35	Weighted κ	0.892	0.749–0.977

ICC were calculated using a two-way random-effects model for absolute agreement based on single measurements [ICC (2,1)]. Quadratic-weighted Cohen’s κ was used for US steatosis grading. Intraobserver reliability was evaluated in a subset of 35 participants. Confidence intervals were estimated using bootstrap resampling at the participant level. CT: Computed tomography; US: Ultrasonography; ICC: intraclass correlation coefficient; CI = confidence interval.

**Table 3:** Training-set performance of ROC analysis for CT attenuation threshold derivation (*n* = 74).

Comparison	HU Cutpoint	Sensitivity	Specificity	Youden Index	AUC	95% CI
Grade 1 vs. ≥ 2	35.50	1.00	0.846	0.846	0.983	0.963–1.000
Grade ≤ 2 vs. 3	28.15	1.00	0.967	0.967	0.995	0.986–1.000

Cutpoints were selected by maximizing the Youden index within the training dataset. Reported performance estimates correspond to the training dataset and do not represent performance in the internal hold-out dataset. HU: Hounsfield units; AUC: area under the curve; CI: confidence interval.

**Table 4:** Distribution of ultrasonographic steatosis grades in the training and internal hold-out datasets.

Dataset	*n*	Grade 1 *n* (%)	Grade 2 *n* (%)	Grade 3 *n* (%)
Training	74	39 (52.7)	21 (28.4)	14 (18.9)
Internal hold-out	31	21 (67.7)	6 (19.4)	4 (12.9)
Full cohort	105	60 (57.1)	27 (25.7)	18 (17.1)

The distributions of ultrasonographic steatosis grades did not differ significantly between the training and internal hold-out datasets (χ^2^ (2) = 2.02, *p* = 0.365).

**Table 5:** Association and agreement between CT-predicted and US steatosis grades in the internal hold-out dataset (*n* = 31).

Measure	Estimate	*p*-Value	Interpretation
Spearman ρ	0.946	<0.001	—
Kendall τ	0.930	<0.001	—
Weighted Cohen κ (quadratic)	0.940	<0.001	Almost perfect

Kappa values were interpreted according to Landis & Koch; κ > 0.80 indicates almost perfect agreement; —: no categorical interpretation applied.

## Data Availability

The original contributions presented in this study are included in the article and Supplementary Materials. Further inquiries can be directed to the corresponding author.

## Supplementary Materials

The following supporting information can be downloaded at https://www.mdpi.com/article/10.3390/diagnostics16182996/s1, Supplementary Figure S1: ROC curves for the two binary classification models derived from the training set (*n* = 74). Solid line: Grade 1 vs. Grade ≥ 2 (AUC = 0.983). Dashed line: Grade ≤ 2 vs. Grade 3 (AUC = 0.995). Optimal cutpoints were selected using the Youden Index. Supplementary Table S1: STARD 2015 checklist.

## Abbreviations

The following abbreviations are used in this manuscript:

CT	Computed Tomography
US	Ultrasonography
HU	Hounsfield units
MASLD	Metabolic Dysfunction-Associated Steatotic Liver Disease
ROC	Receiver operating characteristic
AUC	Area under the curve
BMI	Body mass index
SD	Standard deviation
IQR	Interquartile range
SBP	Systolic blood pressure
DBP	Diastolic blood pressure
